# Modeling of Key Specifications for RF Amplifiers Using the Extreme Learning Machine

**DOI:** 10.3390/mi13050693

**Published:** 2022-04-28

**Authors:** Shaohua Zhou, Cheng Yang, Jian Wang

**Affiliations:** 1 School of Microelectronics, Tianjin University, Tianjin 300072, China; zhoushaohua@tju.edu.cn (S.Z.); ych2041@tju.edu.cn (C.Y.); 2 Qingdao Institute for Ocean Technology, Tianjin University, Qingdao 266200, China

**Keywords:** RF amplifier, temperature characteristics, modeling, ELM

## Abstract

The amplifier is a key component of the radio frequency (RF) front-end, and its specifications directly determine the performance of the system in which it is located. Unfortunately, amplifiers’ specifications degrade with temperature and even lead to system failure. To study how the system failure is affected by the amplifier specification degradation, it is necessary to couple the amplifier specification degradation into the system optimization design. Furthermore, to couple the amplifier specification degradation into the optimal design of the system, it is necessary to model the characteristics of the amplifier specification change with temperature. In this paper, the temperature characteristics of two amplifiers are modeled using an extreme learning machine (ELM), and the results show that the model agrees well with the measurement results and can effectively reduce measurement time and cost.

## 1. Introduction

A radio frequency (RF) amplifier is a key component of the RF front-end [1,2], which has been widely used in various radio systems [3], such as navigation systems [4], radar [5,6] and satellite communication systems [7], mobile communication systems [8], positioning systems [9], etc. Due to different application scenarios and requirements, the above radio systems usually need to work in various environments. Some radio systems even need to work in very harsh environments [10], such as significant variations in temperature and humidity. As a key component of the above radio system, the performance of the RF amplifier is very sensitive to temperature variations. The performance of the radio system will be affected or even fail with the performance specifications of the RF amplifier change with temperature. It may lead to the radio systems not meeting the requirements of the actual application scenario [10]. In [11], although the RF amplifier has not reached the end of its life at this point, its performance specifications have degraded to the point where it cannot meet the minimum requirements for the proper functioning of the radio system. Therefore, the RF amplifier’s performance specifications must be known for a given temperature range to ensure the radio system’s proper functioning.

Much fruitful work has been carried out previously on the study of amplifier specifications versus temperature variations. For example, Gian Piero Gibiino et al. [12] proposed a thermal behavioral model for RF power amplifiers (PAs) directly extracted through baseband and RF measurements of an laterally-diffused metal-oxide semiconductor (LDMOS) PA under pulse-modulated RF input-controlled temperature. The presented model accurately predicts the influence of temperature on quasi-static and dynamic characteristics of an LDMOS PA. Gautam Jindal et al. [13] reported an RF power amplifier behavioral model with temperature effects suitable for predicting the long-term memory effects in standalone and multiple-PA arrays. The model can estimate the PA output power in the presence of self-heating and for a wide temperature range. K. Mohamad Ali et al. [14] focused on the behavioral modeling of thermal effects induced by a power amplifier for system-level simulation to predict better the link budget of RF transmitters for narrow-band radar applications. Finally, Julie Mazeau et al. [15] presented a new electrothermal behavioral model for power amplifiers. This model, implemented into a circuit simulator, allows predicting the impact of the thermal effects in pulsed-RF mode thanks to an envelope transient analysis. The above studies have investigated the relationship between amplifier specifications and temperature in detail and have developed behavioral models. However, the above studies only focus on the amplifier’s thermal effect on its specification, while the actual operating scenarios, where the environment changes (e.g., temperature) also affect the amplifier’s specification. Therefore, knowing the relationship between the amplifier specifications and the change in the external operating environment becomes an urgent problem.

Currently, there are two main ways to obtain the environmental characteristics of amplifier specifications. On the one hand, the specifications of the RF amplifier at different temperatures are obtained by simulation [16,17,18], which is fast. However, the absence of an accurate transistor device model leaves a gap between simulation results and measurement results [19]. However, it is still very effective and convenient for some application scenarios that need to know the trend and range of RF amplifier specifications quickly and qualitatively. On the other hand, another method is to directly obtain the specifications of RF amplifiers at different temperatures by measurement [20]. For example, Li et al. measured the specifications of power amplifiers at 0 °C, 50 °C, 75 °C and 100 °C [20,21], and Kumar et al. measured the specifications of low-noise amplifiers at −40 °C, 27 °C, 75 °C and 100 °C [22]. This method can be more accurate in knowing the specifications of RF amplifiers at different temperatures, but the measurement process takes more time and is also constrained by the measurement environment to measure RF amplifier specifications at each temperature. Therefore, it becomes an urgent problem to know the specification of an RF amplifier at any temperature in each temperature range by measuring a few critical temperature points.

In this paper, temperature characteristic variation measurements are performed for two given RF amplifiers and are based on the data from these measurements. An extreme learning machine (ELM) is applied to model the critical specifications of an RF amplifier concerning temperature. It can be used to predict the variation in the critical specifications of the RF amplifier in each temperature interval. The modeling results show that the accuracy of this model is two orders of magnitude higher than that of the model in the literature [19] for the same number of temperature measurement points, and the selection and distribution of temperature points have little effect on the accuracy of this model.

The organization of this paper is specified as follows. Section 2 gives a brief description of the structure and the measurement setup of the temperature characteristics of the two RF amplifiers. Applying ELM to model the temperature characteristics of two RF amplifiers is described in Section 3. Section 4 provides a detailed discussion and analysis of the modeling results. Finally, the main conclusions of this paper are summarized.

## 2. Designed Circuits and Experiments

**Figure 1:**
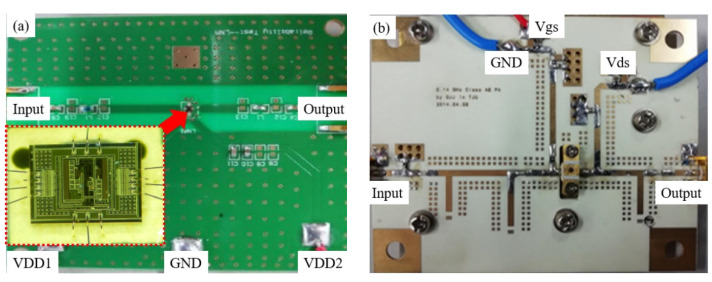
The photograph of the: (**a**) Complementary Metal Oxide Semiconductor low-noise amplifier (CMOS LNA); (**b**) Gallium Nitride class-AB power amplifier (GaN Class-AB PA).

To achieve the goals set in this paper, we chose two RF amplifiers for measurement and modeling. As shown in Figure 1, one is a 50–450 MHz complementary metal oxide semiconductor (CMOS) low-noise amplifier (LNA), and the other is a 0.5–2.1 GHz gallium nitride (GaN) class-AB power amplifier (PA).

Gold wire bonding fixed the LNA to the printed circuit board (PCB). The matching stage of the LNA uses a standard source cascade amplifier with shunt feedback, which is mainly used to improve the noise figure (NF) [23]. The LNA is simulated with frequencies (50~450 MHz, step-size 20 MHz) and temperatures (−40~+90 °C, step size 5 °C). The input power and supply voltage of the LNA is set to be −40 dBm and 1.8 V, respectively.

The 0.5−2.1 GHz GaN Class AB power amplifier consists of a GaN High Electron Mobility Transistor (HEMT) (Cree’s CGH40006P, Durham, NC, USA) fabricated on a Rogers 5880 substrate. The PA was simulated in with a set of frequencies (500~2100 MHz, step-size 20 MHz) and temperatures (−40~+90 °C, step-size 5 °C). Additionally, the input power of the PA is set to be −40 dBm. Besides, the PA’s output power (*P_out_*) at 1.22 GHz is simulated with a set of input powers (0~23 dBm, step-size 1 dBm).

**Figure 2:**
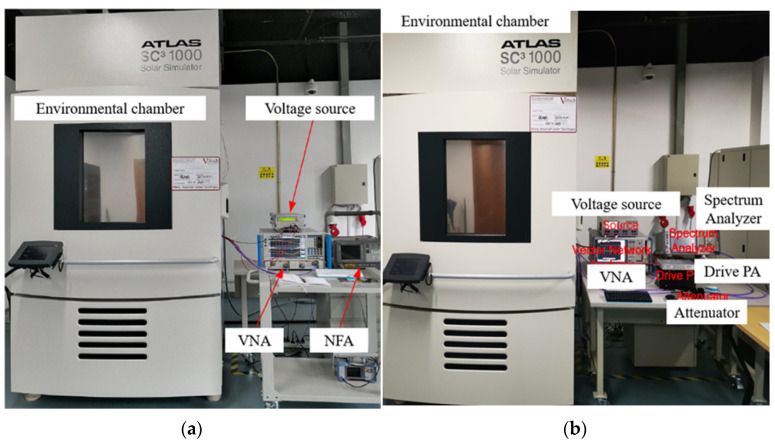
Measurement environment and setup: (**a**) S-parameters and noise figure (NF); (**b**) output power.

As shown in Figure 2, the temperature characterization of the RF amplifier was performed in an environmental chamber (SC^3^ 1000 MHG from Vötsch Industrietechnik (Gie&szlig, Germany)) with a measured temperature range of −40 °C to +90 °C.

The *S*-parameters of PA and LNA were measured by Vector Network Analyzer (VNA, Rohde & Schwarz, Munich, Germany), and the output power (*P_out_*) was measured by the spectrum analyzer (FSV30, Rohde & Schwarz, Munich, Germany) and Rohde & Schwarz Vector Network Analyzer (ZVA24, Rohde & Schwarz, Munich, Germany). In addition, the output power is measured using a drive amplifier ZVA-183-S+ (MiniCircuits, New York, NY, USA) and a 30 dB attenuator. Finally, Agilent Noise Figure Analyzer (NFA) N8975A (Agilent, Santa Clara, CA, USA) measures the NF of the LNA.

The input power, temperature, and frequency ranges are consistent with the simulation settings. Each temperature testing point is stabilized for 30 min before starting the measure to ensure that the temperature of the environmental test chamber has stabilized.

## 3. Modeling Process

### 3.1. Structure of the Model and the Process of Modeling

The lack of an accurate temperature-dependent device model makes it possible that there is always a gap between simulated and measured RF amplifiers [19], but the trend in the simulated and measured results with temperature is similar. Suppose the gap between simulation and measurement results can be eliminated or minimized by calibrating the simulation results with the actual measurement results. In that case, the calibrated “simulation results” can then be used to predict the performance of the RF amplifier at different temperatures over a given temperature range and reduce the number of measurement points required for modeling.

Therefore, to reduce the number of measured temperature points, the simulation data of the RF amplifier is used for modeling the temperature characteristics of the RF amplifier in this paper. The main idea of its modeling is first to establish the relationship between the amplifier performance and temperature variation using the simulation data of the RF amplifier and then calibrate the trained model using the actual measurement data. The greater the number of measurement points used for model calibration, the higher the accuracy of the resulting model.

**Figure 3:**
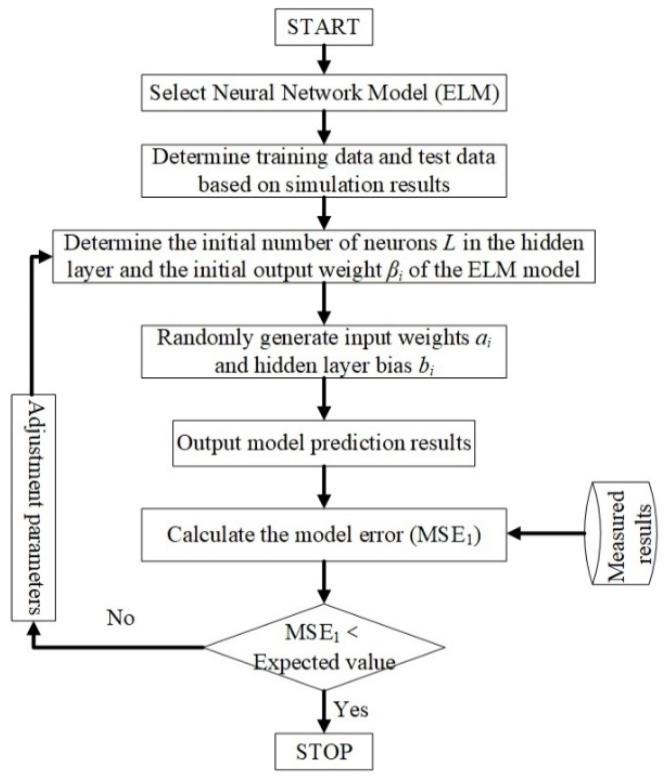
ELM (extreme learning machine)-based modeling flow for RF amplifier temperature characteristics.

ELM is emerging as a new developmental milestone in artificial neural networks. They have been widely applied in microwave millimeter-wave devices/circuits, such as filter design [24], behavior modeling of power amplifiers [25], etc. The relationship between the input and output of an RF amplifier is strongly nonlinear [25], and the ELM has a strong non-linear learning capability [18]. Therefore, ELM was used to model the temperature characteristics of the RF amplifier. The ELM-based modeling process for the RF amplifiers’ temperature characteristics proposed in this paper is shown in Figure 3.

As shown in Figure 3, the process of modeling the temperature characteristics of the RF amplifier is divided into two main parts, one is the training of the model, and the other is the calibration of the model. The main steps are specified as follows.

**Figure 4:**
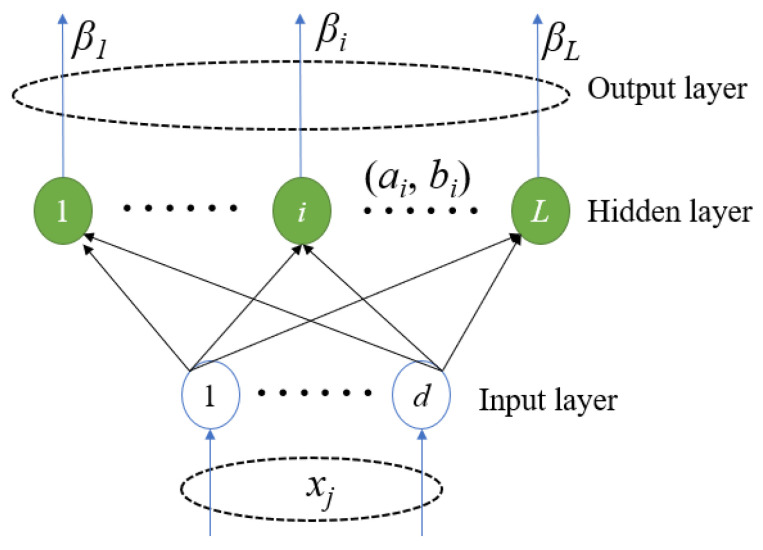
Single-hidden layer feedforward network.

Step 1: Selection of a suitable neural network. As described in the literature [26,27], to establish this nonlinear relationship for the temperature characteristics of the RF amplifier, a single hidden layer feedforward network (SLFN) is used, as shown in Figure 4.

For arbitrary distinct samples (*x_j_*, *t_j_*) (*j* = 1, …, *d*), SLFNs with *L* hidden nodes are mathematically modeled as [27]

(1) $$o_{j}=\sum_{i=1}^{L}\beta_{i}g_{i}(x_{j})=\sum_{i=1}^{L}\beta_{i}G(a_{i}\cdot x_{j}+b_{i})$$

where *x_j_* presents the input power, frequency, and temperature; *t_j_* is the simulation’s S-parameter, NF, and output power; *o_j_* is the model’s output; *β_i_* is the output weight; *a_i_* is the input weight; and *b_i_* is the hidden layer bias. The input weight and hidden layer bias are randomly generated according to the continuous distribution probability [27]. Additionally, *g*(·) is a nonlinear activation function; *G* (·) is the output function of the hidden node [27].

Step 2: Divide the simulation data into training data and test data.
Step 3: Determine the initial number of hidden layer layers (*L*) and hidden layer output weights (*β_i_*) of the model.
Step 4: Randomly generate input weights (*a_i_*) and hidden layer weights (*b_i_*).
Step 5: Output the model results.
Step 6: Calculate the model error (*MSE_1_*).
Step 7: Calibrate of the model using the test results.
Step 8: Compare the model error with the expected value.
Step 9: Determine whether the model is trained or not based on comparing the model error with the expected value.

### 3.2. ELM Training Algorithm

In Section 3.1, SLFNs can be approximated with zero error for *d* samples. Therefore, [25],

(2) $$\sum_{j=1}^{d}\left\|o_{j}-t_{j}\right\|=0$$

(3) $$t_{j}=\sum_{i=1}^{L}\beta_{i}g_{i}(x_{j})=\sum_{i=1}^{L}\beta_{i}G(a_{i},b_{i},x_{j})$$

Equation (2) can be expressed as [25]

(4) Hβ=T

where ***H*** is the hidden layer output matrix (randomized matrix) [28]

(5) $$H=\left[\begin{array}{c} h(x_{1})\\ \vdots \\ h(x_{d}) \end{array}\right]=\left[\begin{array}{ccc} h_{1}(x_{1}) & \cdots & h_{L}(x_{1})\\ \vdots & \vdots & \vdots \\ h_{1}(x_{d}) & \cdots & h_{L}(x_{d}) \end{array}\right]$$

and ***T*** is the training data–target matrix [28].

(6) $$T=\left[\begin{array}{c} t_{1}^{T}\\ \vdots \\ t_{d}^{T} \end{array}\right]=\left[\begin{array}{ccc} t_{11} & \cdots & t_{1n}\\ \vdots & \vdots & \vdots \\ t_{d1} & \cdots & t_{dn} \end{array}\right]$$

The ELM training algorithm can be summarized as follows [28,29].

(1) Randomly assign the hidden node parameters, e.g., the input weights (*a_i_*) and hidden layer weights (*b_i_*), *i* = 1, …, *L*.
(2) Calculate the hidden layer output matrix ***H***.
(3) Obtain the output weight vector.

(7) $$\beta =H^{†}T$$

where ***H***^†^ is the Moore–Penrose generalized inverse of matrix ***H***.

The orthogonal projection method can be efficiently used for the calculation of MP inverse [28]:

If ***H***^T^***H*** is nonsingular,

(8) $$H^{†}={\left(H^{T}H\right)}^{-1}H^{T}$$

if ***HH***^T^ is nonsingular,

(9) $$H^{†}=H^{T}{\left(H^{T}H\right)}^{-1}$$

### 3.3. Error of the Model

Once the first step of this modeling process is completed, it is time for the second step to calibrate the already trained model using the measurement data. The primary process of calibration is by comparing the error (MSE_1_) between the model output (*o_j_*) and the measurement data (*m_j_*), which is expressed as

(10) $${\mathrm{MSE}}_{1}=\frac{1}{n}\sum_{j=1}^{n}{\left(o_{j}-m_{j}\right)}^{2}$$

Suppose the MSE_1_ is less than 10^−3^ or lower. The model is well-calibrated. Conversely, it is necessary to keep adjusting *L* and *β_i_* until the error MSE_1_ meets the requirements. This results in a set of values of *L* and *β_i_* calibrated by the actual measurement data. This set of parameters can then be substituted into the trained model.

## 4. Modeling Results and Discussion

### 4.1. 0.5−2.1 GHz GaN Class AB PA

#### 4.1.1. S21

**Figure 5:**
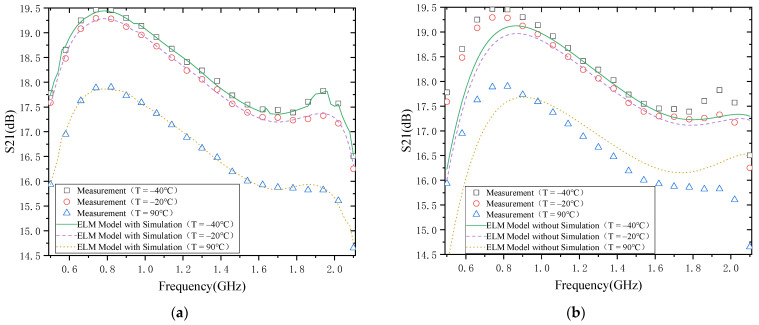
S21: (**a**) with simulation; (**b**) without simulation.

The modeling results of S21 of PA are shown in Figure 5, where Figure 5a,b show the modeling results with and without simulation data. Firstly, from the temperature characteristics of S21, S21 decreases with the increase in temperature. The main reason for the degradation of S21 with the temperature rise is that the two-dimensional electron gas (2DEG) mobility drops. Of course, the increase in temperature will also lead to a decrease in the threshold voltage, which will lead to a rise in S21. However, the gate-source voltage is often much larger than the threshold voltage in the actual circuit to obtain a higher gain. Therefore, the effect of the threshold voltage change on the gain is negligible. It is also consistent with what was reported in the literature [11].

Secondly, from the perspective of model accuracy, the model results of S21 match well with the actual measurement results if the simulation data are used in the modeling process (as shown in Figure 5a). In contrast, if the simulation data are not used in the modeling process of S21 (Figure 5b), there is a large gap between the model results and the actual measurement results. This is mainly because we first used the simula-tion data in the modeling process to describe the trend in the temperature characteris-tics of S21, which is only a little different from the real situation in terms of magnitude. This gap can then be eliminated by calibrating the measured data. Finally, from the point of view of frequency, the variation in S21 at different frequencies is not the same for the same magnitude of temperature change. This indicates that the 2DEG mobility is not only temperature-dependent but also frequency-dependent.

#### 4.1.2. Output Power

**Figure 6:**
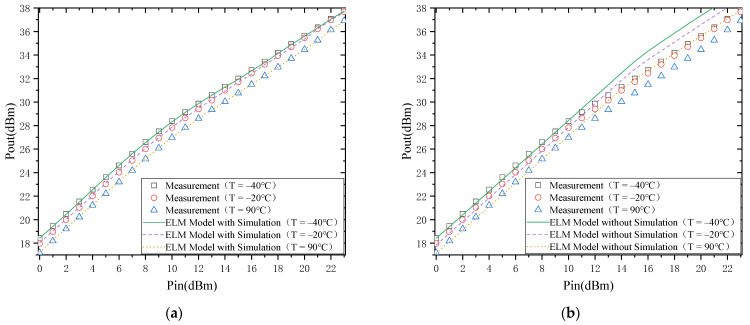
Output power: (**a**) with simulation; (**b**) without simulation.

The modeling results of the output power of the PA are shown in Figure 6. Figure 6a shows the modeling results with simulation data, and Figure 6b shows the results without simulation data. Firstly, from the temperature characteristics of the output power, the output power of the PA decreases with the increase in temperature. This is mainly due to the decrease in 2DEG mobility, increasing source/drain resistance [11]. However, it can also be noted that the drop in output power decreases when the out-put power is close to saturation (when the temperature change is the same).

From the model’s accuracy, it is the same as the modeling result of S21. However, from Figure 6a, there is a better agreement between the model with simulation data and the actual measurement results, while there is a more significant deviation be-tween the model without simulation data and the actual measurement results (Figure 6a). This is also mainly because the trend in the output power temperature characteris-tics can be depicted by the simulation data in the case where simulation data are available.

The above discussion provides a detailed analysis of the relationship between S21 and temperature and output power and temperature. Next, the effect of the model will be explicitly discussed in terms of the results of the model. As shown in Figure 5 and Figure 6, the ELM model based on the simulated data agrees well with the measured results. However, at the same time, there is a significant deviation between the ELM model without simulated data and the measured results. This is mainly because the trend between simulated and measured data is the same, and there are only some differences in the values. Therefore, the trend in the temperature characteristics of the PA is first obtained from the simulated data and then calibrated by the measured data. As a re-sult, the model based on the simulated data matches the actual trends and values measurements. In contrast, the modeling is based on measurement data, where the number of measured temperature points is very small, and the behavior of many tem-perature points is not learned, so there is a significant error between the model output and the actual measurement results.

To further compare the differences between the modeling method in the literature [19] and the modeling method proposed in this paper, the errors of the two modeling methods are compared at different numbers of temperature measurement points, as shown in Table 1. Table 1 shows the results compared to the modeling approach in the literature [19]. The modeling method proposed in this paper has the following ad-vantages: first, it is less sensitive to the temperature measurement points; second, the model accuracy is two orders of magnitude higher with the same number of measure-ment temperature points; and third, the number of required temperature measurement points is less with the same model accuracy requirement.

### 4.2. 50−450 MHz CMOS LNA

#### 4.2.1. S21

**Figure 7:**
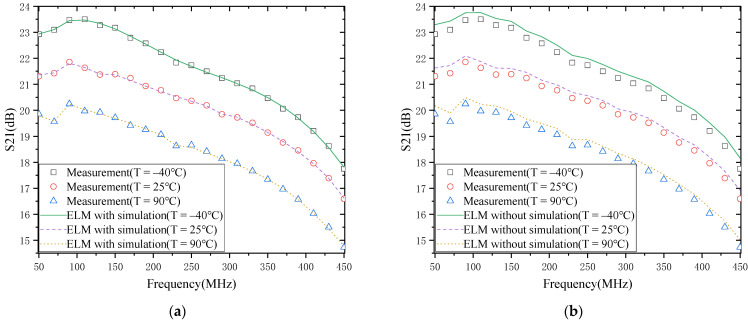
S21 of the LNA: (**a**) with simulation; (**b**) without simulation.

The modeling results of S21 of LNA are shown in Figure 7. Figure 7a,b show the modeling results with simulation data and without simulation data. First, in terms of the temperature characteristics of S21, S21 of LNA decreases with the increase in temperature. This phenomenon is mainly because the rise in temperature causes the carrier mobility to decrease and thus the trans-conductivity to decrease. It can also be noted that when the temperature change is the same at different frequencies, the magnitude of S21 change is not the same. For example, when the temperature increases from −40 °C to 90 °C, the S21 distribution at 70 MHz and 450 MHz decreases by 3.54 dB and 3.02 dB. This also shows that the carrier mobility is temperature-dependent and frequency-dependent. Second, in terms of the model’s accuracy, it is evident that the model with simulation data agrees well with the actual measurement results, while the model without simulation data has a significant deviation from the actual measurement results. This indicates that a better modeling result can be achieved using the simulation data with the same number of measurement points and a smaller number of measurement points.

#### 4.2.2. The NF of the LNA

**Figure 8:**
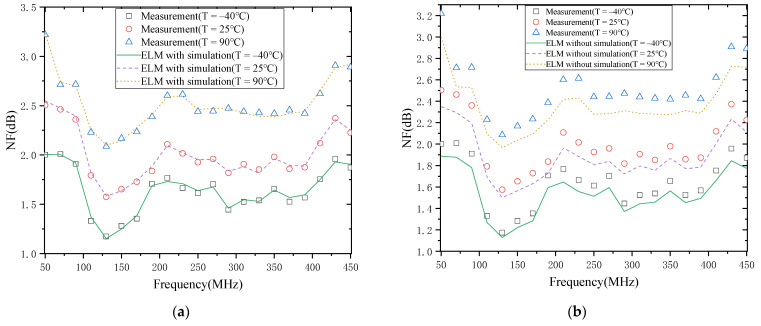
NF of the LNA: (**a**) with simulation; (**b**) without simulation.

The results of modeling the temperature characteristics of the NF of the LNA are shown in Figure 8. Figure 8a shows the modeling results with simulation data, and Figure 8b shows the results without simulation data. From Figure 8a, the NF increases with the temperature increase. This is mainly because the carrier mobility decreases whenever the temperature increases, making the trans-conductivity decrease. In turn, the reduction in trans-conductivity will lead to a rise in NF [30]. It means that the NF of LNA increases with the temperature rising and is consistent with the results reported in [22].

In addition, it can be found that the change in NF is different at different frequencies when the magnitude of temperature change is the same. For example, when the temperature increases from −40 °C to 90 °C, the NF increases by 1.22 dB and 0.74 dB at 50 MHz and 270 MHz, respectively, which shows that the carrier mobility increases are not only temperature-dependent but also frequency-dependent. Then, from the results in Figure 8a,b, the model with simulation data has a smaller gap and better agreement with the actual measurement results than the model without simulation data, achieving the expected modeling effect. This shows that using simulation data to describe the trend in NF temperature characteristics first, and then calibrating it with actual measurement data, is feasible. Furthermore, this approach can significantly reduce the time and cost of measurement.

The above results are a specific analysis and discussion of the S21 and NF of the LNA versus temperature. The following results of modeling S21 and NF of LNA are discussed and analyzed in detail. As can be seen in Figure 7 and Figure 8, the modeling results of LNA agree well with the actual measurement results. The main reason is that the model accuracy in the literature [19] depends on the number of measured temperature points, and with a small number of measured points, the behavior of many temperature points is not learned, resulting in poor model accuracy. In contrast, the modeling method proposed in this paper first uses simulation data to obtain a rough model of the temperature characteristics of the LNA and then calibrates this rough model with actual measurement data. In this way, a better model accuracy can be achieved based on the data of a small number of measurement points.

Similarly, to compare with the modeling approach in the literature [19], the accuracy of the two modeling approaches is compared in this paper (as in Table 2). Table 2 shows the following advantages over the modeling approaches in the literature [19]: first, it is less sensitive to temperature measurement points; second, the accuracy of the model is two orders of magnitude higher with the same number of measurement temperature points; third, the number of measurement temperature points required is less with the same model accuracy requirement.

This paper verifies the effectiveness of the proposed ELM-based RF amplifier modeling method by taking a GaN PA of 0.5−2.1 GHz and a CMOS LNA of 50−450 MHz as examples. It is also compared with the modeling method in the literature [19]. The modeling method in the literature [19] is mainly based on measurement data and uses ELM for modeling. This modeling approach needs to rely on a relatively large number of measurement points. If the number of measurement points is too small, the model accuracy will be very poor because the model has not learned the trends in many temperature points. In contrast, the modeling method proposed in this paper mainly uses simulation data to build a coarse model first and then calibrates the coarse model using measurement data, and this modeling method only needs a few measurement data to achieve a high model accuracy.

From Table 1 and Table 2, the modeling method proposed in this paper has the following advantages, both for modeling PA and for modeling LNA: first, it is less sensitive to temperature measurement points; second, the model accuracy is two orders of magnitude higher than the model accuracy in [19] with the same number of measured temperature points; third, only three temperature measurement points are required with the same model accuracy requirement, while eight temperature measurement points are required in [19].

In summary, the modeling method proposed in this paper can not only model the temperature characteristics of the RF amplifier but also significantly reduce the number of measured temperature points while ensuring the model’s accuracy.

Finally, it should be noted that the data used for modeling in this paper are from static simulation/measurement points. Additionally, the modeling of the dynamic behavior of amplifiers is currently receiving more and more attention from the industry. The ELM model used in this paper has not been verified as to whether it is suitable for modeling the dynamic behavior of amplifiers, which is currently receiving a lot of attention. However, the authors believe that the ELM model used in this paper can be used to model amplifiers’ dynamic behavior. This is because the ELM model used in this paper is a behavioral-level black-box model, which only cares about the input and output variables and data, not about the specific physical mechanism. Therefore, only the input and output variables and data need to be known, and the ELM model can still be used for modeling. All that remains is adjusting and optimizing the model parameters until the model meets the desired requirements.

## 5. Conclusions

In this paper, an ELM-based modeling method for the temperature characteristics of RF amplifiers is proposed. Additionally, the effectiveness of this modeling method is verified by taking PA and LNA as examples. The results show that the modeling method proposed in this paper can realize the modeling of RF amplifier temperature characteristics and significantly reduce the number of measured temperature points with very low sensitivity to the temperature points while ensuring the model accuracy. Compared with the existing modeling methods, the modeling method can effectively reduce the measurement time and lower the cost. Furthermore, the model proposed in this paper can characterize the static characteristics of RF amplifiers and be extended to model the behavior of dynamic characteristics of RF amplifiers or other devices.

## Figures and Tables

**Table 1:** Comparison results of models in different cases for power amplifiers (PA).

Number and Distribution of Measured Temperature Points		MSE	
No.	Temperature (℃)	This Work	Ref. [19]
3	−40; 25; 90	9.2317 × 10^−3^	4.7517 × 10^−1^
3	−40;−20; 90	9.3243 × 10^−3^	1.4372 × 10^0^
5	−40; −10; 25; 60; 90	9.1524 × 10^−4^	9.9561 × 10^−2^
5	−40; −5; 0; 15; 90	9.2037 × 10^−4^	3.3107 × 10^−1^
6	−40; −10; 15; 40; 65; 90	8.5972 × 10^−4^	9.6521 × 10^−2^
6	−40; −20; 0; 70; 80; 90	8.6113 × 10^−4^	2.8317 × 10^−1^
7	−40; −20; 0; 25; 50; 70; 90	8.3821 × 10^−4^	9.4621 × 10^−2^
7	−40; −30; −20; 50; 60; 75; 90	8.4981 × 10^−4^	2.5237 × 10^−1^
8	−40; −30; −10; 10; 30; 50; 70; 90	8.1681 × 10^−5^	9.2386 × 10^−3^
8	−40; −35; −5; 15; 20; 25; 85; 90	8.2234 × 10^−5^	2.3025 × 10^−2^
9	−40; −25; −10; 5; 25; 45; 60; 75; 90	7.8475 × 10^−5^	9.0274 × 10^−3^
9	−40; −5; 10; 15; 35; 60; 65; 85; 90	8.0521 × 10^−5^	2.0679 × 10^−2^

**Table 2:** Comparison results of models in different cases for LNA.

Number and Distribution of Measured Temperature Points		MSE	
No.	Temperature (℃)	This Work	Ref. [19]
3	−40; 25; 90	8.8737 × 10^−3^	4.4758 × 10^−1^
3	−40; 40; 90	9.0481 × 10^−3^	2.4478 × 10^0^
5	−40; −5; 25; 55; 90	8.6943 × 10^−4^	9.5745 × 10^−2^
5	−40; 0; 25; 30; 90	8.7042 × 10^−4^	5.6612 × 10^−1^
6	−40; −15; 10; 35; 60; 90	8.3569 × 10^−4^	9.3036 × 10^−2^
6	−40; −20; −5; 20; 50; 90	8.4327 × 10^−4^	4.8756 × 10^−1^
7	−40; −15; 5; 25; 45; 65; 90	7.9783 × 10^−4^	9.1069 × 10^−2^
7	−40; −30; 0; 10; 40; 70; 90	8.1678 × 10^−4^	4.2132 × 10^−1^
8	−40; −20; 0; 20; 40; 60; 80; 90	7.5237 × 10^−5^	8.8607 × 10^−3^
8	−40; −25; −15; 0; 15; 35; 40; 90	7.6654 × 10^−5^	6.8072 × 10^−2^
9	−40; −20; −5; 10; 25; 40; 55; 70; 90	7.0612 × 10^−5^	8.5492 × 10^−3^
9	−40; −35; −5; 0; 15; 40; 65; 75; 90	7.1357 × 10^−5^	6.5627 × 10^−2^
